# Card of the Committee on Prize Essays of the American Medical Association

**Published:** 1855-12

**Authors:** 


### EDITORIAL.

*Card of the Committee on Prize Essays of the American Medical Association.*—At a meeting of the American Medical Association held in Philadelphia, May 1855, the undersigned were appointed a committee to receive voluntary communications on Medical Subjects, and to award prizes in accordance with the regulations of that body.

Each communication intended to compete for a prize must be addressed to the chairman of the committee at Ann Arbor, Michigan, before March 20th, 1856, and must be accompanied with a sealed packet containing the name of the author, and marked exteriorly by a sentence or motto corresponding with one upon the essay; which packet will not be opened unless the essay belonging to it is successful in obtaining a prize.

Unsuccessful papers will be returned on application after the adjournment of the meeting of the Association at Detroit in May next.

A. B. Palmer, M. D., (chairman), S. Denton, M. D., A. B. Terry, M. D., A. Sager, M. D., S. H. Douglass, M, D., C. L. Ford, M. D., E. Andrews, M. D.

---

WASHINGTON, Nov. 30, 1855.

DR. N. S. DAVIS :

DEAR SIR,—At the last Annual Meeting of the National Medical Association, a committee was authorized—of which I was appointed chairman, and associated with Doctors Francis Condie, of Pennsylvania, and Grafton Tyler, of the District of Columbia' to report what measures should be adopted to remedy the evils existing in the present system of holding Coroner's inquests.

This is a subject which comes home to every one of us, and it is one in which are involved the gravest interests, and as such should commend it to the consideration of every medical man. It is highly desirable to obtain any data re lating to this subject such as the statutory provisions, the forms of procedure, the fees paid to medical witnesses; and it is desirable to ascertain what is the tenure of, and what are the qualification for the office of Coroner.

Any facts or suggestion will be most thankfully received and duly acknowledged.

I am respectfully your Ob't. Ser't.

A. J. SEMMES, M. D.

